# The Potential of Nanomedicine to Unlock the Limitless Applications of mRNA

**DOI:** 10.3390/pharmaceutics14020460

**Published:** 2022-02-21

**Authors:** Laura Taina-González, María de la Fuente

**Affiliations:** 1 Nano-Oncology and Translational Therapeutics Group, Health Research Institute of Santiago de Compostela (IDIS), SERGAS, 15706 Santiago de Compostela, Spain; laura.taina@rai.usc.es; 2 Universidad de Santiago de Compostela (USC), 15782 Santiago de Compostela, Spain; 3 Cancer Network Research (CIBERONC), 28029 Madrid, Spain; 4 DIVERSA Technologies, 15782 Santiago de Compostela, Spain

**Keywords:** mRNA, LNPs, vaccine, COVID-19, nanomedicine, nanotechnology, gene therapy, CRISPR, cancer, protein replacement

## Abstract

The year 2020 was a turning point in the way society perceives science. Messenger RNA (mRNA) technology finally showed and shared its potential, starting a new era in medicine. However, there is no doubt that commercialization of these vaccines would not have been possible without nanotechnology, which has finally answered the long-term question of how to deliver mRNA in vivo. The aim of this review is to showcase the importance of this scientific milestone for the development of additional mRNA therapeutics. Firstly, we provide a full description of the marketed vaccine formulations and disclose LNPs’ pharmaceutical properties, including composition, structure, and manufacturing considerations Additionally, we review different types of lipid-based delivery technologies currently in preclinical and clinical development, namely lipoplexes and cationic nanoemulsions. Finally, we highlight the most promising clinical applications of mRNA in different fields such as vaccinology, immuno-oncology, gene therapy for rare genetic diseases and gene editing using CRISPR Cas9.

## 1. Prospects and Limitations of mRNA

The first isolation of messenger RNA (mRNA) was published in *Nature* in 1961 [1,2], starting the path towards a full understanding of this molecule [3]. The potential to synthetically produce mRNA from DNA in the 1980s, i.e., in vitro transcribed mRNA (IVT mRNA), opened a broad spectrum of preclinical applications [4]. Since then, multiple efforts have been performed to understand its mechanism of action and the path towards developing mRNA-based drugs. Therapeutic mRNA’s potential lies in its capacity to induce the expression of proteins [4,5] for preventing or altering a particular disease state. mRNA therapeutics hold many opportunities, which can depend on the targeted cells, organ selective accumulation and encoded protein of interest. Nowadays, however, two main approaches are considered when using mRNA, both of which will be reviewed and explained in depth. The first is dendritic cell (DC) targeting, so as to achieve immune activation. The second is to exploit the natural capacity of nanoparticles to accumulate in the liver and use it to endogenously produce therapeutic proteins. 

Importantly, mRNA has several advantages over other gene therapies such as DNA or pDNA which make this molecule more translational in terms of pharmaceutical properties: (i) mRNA does not need to reach the nucleus of the cell as opposed to DNA, resulting in more efficient delivery; (ii) mRNA does not integrate into the genome of the host cell, a fact associated with risk of mutagenesis; (iii) mRNA can be synthesized in the lab with fairly easy protocols, following scalable processes in agreement with GMP regulations; and (iv) mRNA sequences can be easily modified and updated, which is an important fact to consider in vaccinology (i.e., when mutations of the target protein occur). These advantages are also important when comparing mRNA technology to protein delivery technology, which normally comes with short half-lives and expensive and tedious industrial processes [4,5,6].

However, despite all these important advantages, two major drawbacks had been holding up the clinical development of this technology: its high instability and its potential immunogenicity. On one hand, if naked mRNA were administered, ribonucleases (RNA-ases) present in extracellular fluids would quickly degrade it; on the other hand, exogenous RNA molecules would be recognized by activating toll-like receptors (TLR) and trigger immunological responses [7]. Moreover, naked mRNA is not capable of crossing lipid bilayers to reach its target. To overcome these limitations and to improve the pharmacokinetic and pharmacodynamic properties of naked mRNA, two main strategies have been proposed to date: the introduction of chemical modifications on the sequence and the use of a delivery vehicle, which will be discussed in more detail in the following sections.

### 1.1. Structure and Chemical Modifications of mRNA

From a chemical point of view, mRNA is a single-strand biopolymer composed of nucleotide subunits linked by phosphodiester bonds. Each nucleotide is composed of a ribose sugar, a phosphate group and a nitrogenous base (cytosine, C, guanine, G, adenine, A and uracil, U). The mRNA sequence has a 7’methyl guanosine residue (m7G) at the 5’ cap and a poly(A) tail at the 3’ end. As other oligonucleotides, mRNA forms secondary structures thanks to the hydrogen bonding between complementary nucleotides, which is important for its stability [8]. Similarly, another promising technology, self-amplifying RNA (saRNA), is presented when the mRNA molecule is engineered to include an RNA virus genome [4], acquiring the capability to self-replicate and encode for multiple protein copies. This platform allows not only for the maintenance of the aforementioned mRNA’s advantages, but also shows a further benefit by requiring much lower doses [9]. For instance, its pharmacological activity can be maintained for up to two months, further increasing the potency and reducing manufacturing costs of both excipients and RNA molecules, potentially allowing for even higher-speed development processes. The fact that the saRNA molecule is longer, however, could result in a loss of stability and increased risk of immunogenicity [10]. A recently published review on saRNA vaccines further explains and comments the potential of these platforms [11].

As previously mentioned, one of the main advantages of mRNA is that it can be easily synthesized in a laboratory. This process can be performed following two main strategies. The first, which is very commonly used, is based on the in vitro transcription of a linearized pDNA. The second is by making use of PCR, using a template with a bacteriophage promoter, a 5’ UTR, an open reading frame or ORF, a 3’ UTR and a poly[d(A/T)] sequence [5]. For saRNA, the introduction of a replicase prior to the antigen sequence would allow the self-replication of the RNA once administered [11,12].

As introduced, mRNA can be subjected to chemical modifications to achieve increased in vivo stability. Some reported modifications refer to changes in the m7G residue which would prevent the mRNA from being rapidly degraded by RNA-ases and its recognition via the immune system [13,14]. In this sense, recent advances in 5’cap analogues for enhancing mRNA expression include ARCAs (anti-reverse cap analogues) and Beta-S-ARCAs, as well as the incorporation of a 2S analogue [15]. Other modifications to enhance stability are related to the incorporation of longer PolyA tails, which would lead to a change in the secondary structure of mRNA [16,17]; substitutions using synthetic nucleotides such as N1-methyladenosine or N6-methyladenosine instead of natural adenosine, cytidine with 5-methylcytidine or uridine with pseudouridine [18,19]. Improvements in the purification processes and removal of secondary products from the in vitro transcription process have shown to reduce immunogenicity [20]. For further information regarding the chemical modification of RNA, we invite the reader to consult specialized reviews [21,22].

In line with the subject of this review, it should be mentioned that marketed COVID-19 vaccines, described in detail in Section 2.1, have modified RNA sequences [23,24]. However, it is worth noting that this first strategy is not always used, as it can be seen in the available data published for ongoing clinical trials (Table 1 and Tables 2–5). Furthermore, considering the disappointing recently published CureVac mRNA vaccine results [25], described in Section 2.3, which uses unmodified mRNA, it seems plausible that this strategy is potentially needed for achieving good and translational in vivo results. As will be further detailed in Section 2.3, unmodified mRNA sequences are often related to higher reactogenicity, increasing tolerability issues as compared to modified sequences. The second strategy—referring to the use of a delivery vehicle able to efficiently associate, protect and deliver the intact mRNA molecule to its target—is discussed next.

## 2. Delivery Vehicles for mRNA

### 2.1. Lipid Nanoparticles (LNPs), the Delivery Vehicles behind COVID-19 mRNA Vaccines

The Moderna and BioNTech/Pfizer mRNA vaccines were first marketed under emergency authorization in December 2020. Both vaccines were developed in less than a year, proving the potential of mRNA technology to be quickly adapted to emergency situations. These vaccines are composed of SARS-CoV-2 spike protein (S) [23,24] loaded into LNPs and have not only meant a breakthrough in terms of design and manufacturing speed, but also in terms of efficacy. The BioNTech/Pfizer vaccine exhibited a 95% efficacy in phase 3 of clinical trials [26], and more recently, another study was released showing that efficacy was maintained over a period of 6 months, irrespective of previous SARS-CoV-2 infections [27]. For its part, the Moderna vaccine’s phase 3 clinical trial results showed an efficacy of 94.1% [28]. A recent published paper compared the immune response kinetics between mRNA vaccines with the Janssen vaccine, concluding that mRNA vaccines elicit a high initial peak antibody response which decreases after 8 months, while the Janssen vaccine had stable but lower antibody levels over the same period of time [29]. With regards to this, clinical data on vaccine safety and efficacy in children from 5 to 11 [30] and in adolescents [31,32] is available now, increasing the application of this vaccine technology to the younger population as well. Regarding variants, up until the appearance of Omicron, available data suggested that mRNA vaccines protect against all the other variants, including Delta [33,34,35]. However, in regard to this new variant, contradictory data have been shown. On one hand, this study—not yet peer-reviewed—showed a decrease in antibody levels against Omicron [36]. Similarly, in another study which used antibodies from vaccinated and convalescent people to study the effectiveness against all variants, found that the neutralization of Omicron was much lower than of any other variant [37]. On the other hand, there are also data supporting the claim that T-cell immunity is still effective against Omicron [38]. 

None of these achievements would have been possible without nanotechnology, defined as the production and application of structures, devices and systems by controlling the shape and size of materials at a nanometer scale. In recent years, nanotechnology applied to medicine has been referred to as nanomedicine [39,40]. The term “nanomedicine” thus comprises formulations in the nanometer scale (i.e., nanoformulations) composed of organic materials such as lipids or polymers, or inorganic materials such as metals, that can be used to deliver small molecules or biomolecules, namely proteins, peptides or nucleic acids. Nanomedicine can be used for therapeutic purposes [41], but it also has an application in diagnostics and regenerative medicine [42] With respect to the specific application of nanomedicine in gene delivery, all the research performed in the last 30 years has resulted in safe and effective delivery platforms for nucleic acids, such as antisense oligonucleotides (ASOs), small interfering RNAs (siRNA), DNA and more recently mRNA, using different types of strategies such as Gal-NAc conjugates, and different delivery vehicles such as Adeno-Associated Virus (AAD) and more recently LNPs, as recently reviewed in this article [43]. Despite this great effort, at present there is only one nanoformulation in the market for siRNA delivery, Onpattro^®^; however, many others are undergoing clinical trials, and more genetic nanomedicines are expected to be seen in the near future [43]. In this term, the approval of Onpattro^®^ for hereditary transthyretin-mediated (hATT) amyloidosis in 2018 meant a huge breakthrough in nanoparticle design; LNPs, later used for the development of mRNA vaccines, were marketed for the first time. Therefore, these nanoparticles have been key for the development of the two marketed COVID-19 vaccines and are today the most advanced technology in mRNA delivery. The origin of LNPs lies in the discovery of liposomes, bilayer lipid structures which were firstly described in 1965 by Banghman, setting up the basis of model membrane systems [44]. A few years later, Gregoriadis et al. established the idea that these lipidic vesicles could be used for drug-delivery purposes, opening a door towards a full development of the liposome field [45,46,47], and later LNPs [48]. Other lipidic-based delivery vehicles have also been a matter of research, whose most recent advancements will be discussed in Section 2.2. In this next section, we will describe in depth the composition, structure, manufacture and stability considerations of LNPs. 

#### 2.1.1. Unraveling LNP Composition

LNPs used today have a very well-established composition based on a mixture of lipids whose ratios, types and structures are carefully defined. Major constituents are (i) an ionizable lipid, (ii) structural and helper lipids and (iii) PEG-lipid.

**Figure 1:**
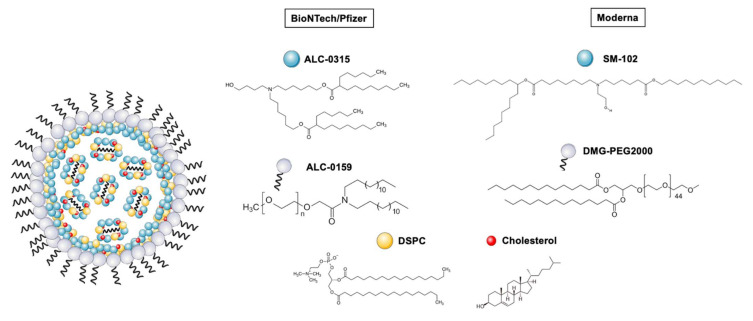
Structure and composition of the authorized COVID-19 mRNA formulations.

**Figure 2:**
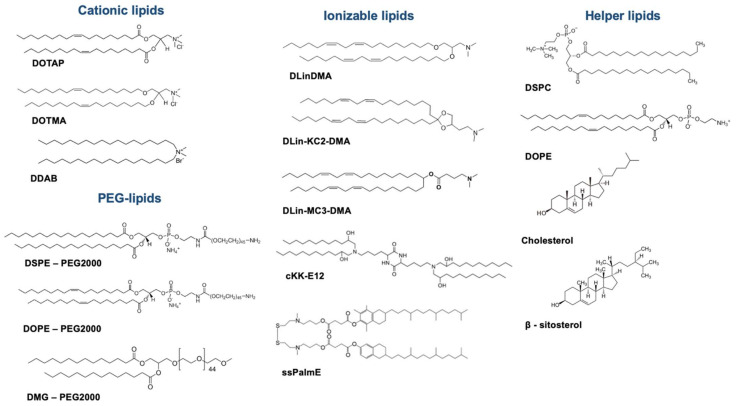
Chemical structure of some LNPs’ components.

**Figure 3:**
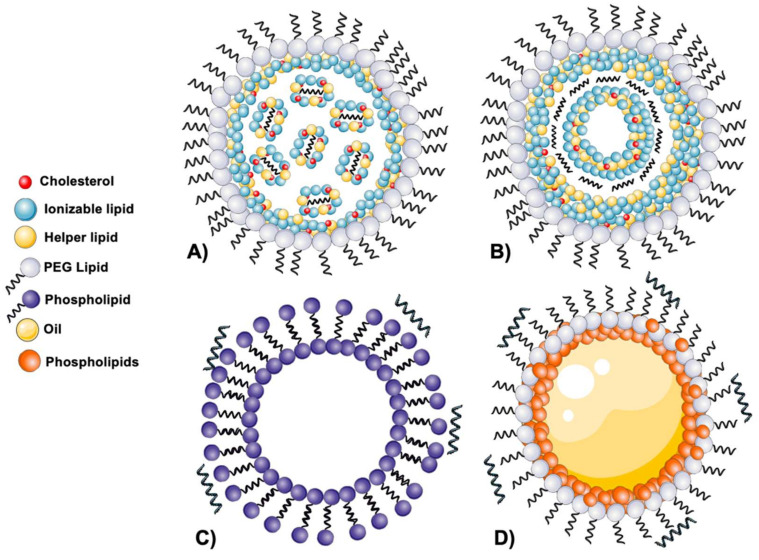
Lipid delivery vehicles for mRNA delivery. (**A**) Lipid nanoparticles (LNPs), structure proposed [87]; (**B**) Lipid nanoparticles (LNPs), structure proposed. Reproduced from Viget et al. [86] which is licensed under a Creative Commons Attribution-(CC BY 4.0) International License (http://creativecommons.org/licenses/by/4.0/); (**C**) Lipoplexes (LPX) and (**D**) cationic nanoemulsions (.NEs).

For each of the vaccines, detailed compositions are described here. The BioNTech/Pfizer LNP composition (ionizable lipid: phospholipid: cholesterol: PEG-lipid) is ALC-0315:DSPC:Chol:ALC-0159 (molar ratio 46.3:9.4:42.7:1.6), with 30 μg of mRNA dose. Moderna LNP composition is SM-102:DSPC:Chol:DMG-PEG (molar ratio 50:10:38.5:1.5) with 100 μg mRNA dose [49]. Each of these constituents possesses a crucial function in the structured nanoparticle, described in detail below. A schematic representation of the composition of LNPs for both vaccines is shown in Figure 1, and chemical structures of lipidic components are shown in Figure 2. The detailed structure hypothesis is explained in Section 2.1.3 and represented in Figure 3.

(i) Ionizable lipids are possibly the most innovative component of LNPs, having represented a huge breakthrough in RNA delivery. Their mechanism of action is based on complexing the nucleic acid by electrostatic interactions. Several properties, such as the charge or lipid shape, as well as the protein corona formation, have been described as important factors to consider when understanding structure–activity relationship studies, and thus, the design of new ionizable lipids. 

On behalf of the charge, prior to the use of ionizable lipids, permanently positively charged lipids, such as DOTAP, DOTMA or DDAB, were of preference. However, the positive surface charge contributed to low stability due to aggregation phenomena, and had toxicity issues [50]. By contrast, today the most commonly used lipids are ionizable lipids: pH-titratable lipids which are positively charged only at acidic pH, allowing the formation of complexes with nucleic acids during the formulation process, but are neutral at pH7’4, overcoming the inconveniences of cationic lipids, and as a result, allowing for longer circulation times, increasing accumulation in targeted tissues and promoting better tolerability [51,52]. Their use, together with the optimization of manufacturing processes, as described in Section 2.1.2, allows the mRNA to be encapsulated with high efficiency inside the nanoparticle, in contrast to what is reported for conventional cationic lipoplexes or cationic nanoemulsions, described in Section 2.2. Today, although the use of these lipids is predominant for new LNP design and SAR studies, permanently positive cationic lipids are still widely used, especially when considering other lipid-based technologies.

The lipid shape—which comes hand in hand with the degree of the lipid chain’s degree of unsaturation—also seems to play an important role in promoting endosomal escape. Heyes et al. proved that when increasing the number of instaurations, the efficiency of delivery increases dramatically due to the formation of H11 hexagonal phase in the particle that promotes fusogenicity with the cell membrane [53]. Semple et al. further discussed this by hypothesizing that once in the endosome, lipids would be protonated thanks to the acidic environment. This in turn would facilitate the interaction with the anionic phospholipids of the endosomal membrane, disrupting it and liberating the cargo [53,54].

The design of new lipids has been evolving ever since the first proof of concept for ApoB gene silencing using DLinDMA lipid in hepatocytes was achieved [55]. This research led to the discovery of DLin-KC2-DMA, which demonstrated promotion of increased in vivo activity at siRNA doses of 0.01 mg/kg in rodents and 0.1 mg/kg in non-human primates in silencing factor VII [54]. On its part, DLinMC3DMA (i.e., MC3) lipid, considered even today a gold standard for LNP formulation development, was first discovered after carrying out a systematic screening to improve gene silencing in vivo, which also concluded that the optimum pKa for hepatic delivery should stand between 6.2 and 6.5 [56]. This lipid would later be commercialized with Onpattro^®^ in 2018 [57].

As opposed to cationic lipids, a specific uptake mechanism has been postulated for these ionizable lipids closely related to the formation of a protein corona enriched in Apolipoprotein E (ApoE), a well-known phenomenon driving the nanoparticle’s fate and biodistribution upon administration [58], as firstly disclosed by Akin et al. [59]. This work, which compared LNPs formulated with the ionizable lipid DLinKC2DMA (iLNPs) versus cationic-lipid LNPs (cLNPs), refers to an ApoE-dependent mechanism by which iLNPs would be efficiently endocytosed by hepatocytes via LDL receptor, mediating accumulation in this organ. Further studies suggested that the main mechanism driving the endocytosis for ionizable DLinMC3DMA-based LNPs were both clathrin-mediated endocytosis as well as macropinocytosis [60]. On this note, the most recent publication on LNP structure indicated that upon binding DLinMC3DMA-based LNPs with ApoE, a rearrangement is induced in the structure of the nanoparticle, which could drive the nanoparticle’s fate [61]. More recently, other studies with mRNA showed that efficient LNP–mRNA transfection could also rely on an early narrow endosomal escape window before lysosomal sequestration or exocytosis [62]. Furthermore, another study showed a link between exogenously administered mRNA and extracellular vesicles (EVs) after observing mRNA present in these EVs at 1:1 ratio, concluding that mRNA should be neutrally charged by the ionizable lipid to promote endosomal escape [63].

As a step forward in lipid design, a subtype of ionizable lipids was presented in 2008, commonly called lipidoids, with a dendrimer-like structure [64] and with the advantage of a straightforward synthesis protocol. Later, Dong et al. reported the synthesis of cKK-E12, a lipidoid capable of silencing hepatocytes with the highest potency in rats, mice and non-human primates reported until that point [65]. 

Research on new ionizable lipids has also been focused on obtaining more biocompatible and biodegradable lipids, classified as new-generation lipids. In this term, Maier et al. showed that including an ester bond in the alkyl chains improved tolerability and retained potency, as compared to MC3 [66]. Other studies have also reported better cell viability in vitro by including disulfide bonds [67], which led Akita et al. to firstly synthesize ssPalmE, a lipid which incorporates a tocopherol-like moiety in its structure [68].

(ii) Structural and helper lipids are also constituents of LNPs, playing an important role in terms of stability and fusogenicity [69]. These lipids are mainly phospholipids (such as DOPE, DSPC, DEPC, DSPE) and cholesterol, which forms the main skeleton of LNPs and might aid in endosomal escape.

Phospholipids are a key component, possibly due to their nature mimicking biological membranes. However, not all phospholipids seem to be equally efficient. It has been reported that the type of structures formed by phospholipids is an important factor driving fusogenicity. In fact, it seems that lipid species such as unsaturated phosphatidylethanolamines (PEs) adopt non-bilayer structures, which in turn are less stable, driving the disruption of the LNPs and inducing fusion with the cell membrane and the consequent release of the cargo inside the endosome [54,70]. Therefore, the structure and unsaturation of phospholipids also seem crucial to improve endosomal escape. When in vivo biodistribution is considered, the helper lipid structure also seems to play a major role. In this regard, a recent study which aimed to systematically screen a library of 96 LNP compositions found that DOPE-containing LNPs preferentially accumulated in the liver, as opposed to the DSPC-containing LNPs. Furthermore, authors reported that DOPE-containing LNPs also exhibited higher ApoE-binding affinity, which also correlated to higher liver-protein expression [71]. On the other hand, cholesterol has been shown to give stability to LNPs and improve efficacy by enhancing membrane fusion [72,73]. Patel et al. have also investigated different cholesterol analogues and their function in enhancing intracellular delivery of mRNA, concluding that the type and flexibility of the alkyl tail present in the sterol ring can affect transfection efficiency [74].

(iii) Polyethilenglycol (PEG) is a widely known polymer that has been described and previously used in liposomes and other types of nanoformulations. It seems to play a main role in prolonging circulation time by reducing macrophage phagocytic system (MPS) interactions, improving colloidal stability and preventing the formation of the protein corona [53,75,76]. PEGylated lipids typically included in these formulations consist of two domains, a hydrophilic PEG polymer conjugated to a hydrophobic lipid branch. In LNPs, the hydrophilic domain is located towards the exterior surface of the particle and the lipid branch towards the interior. Additionally, LNP properties can be tuned by modulating the PEG-lipid/total lipid content ratio. For instance, it is known that smaller LNPs are generated at higher PEG-lipid ratios—a matter that is further described in Section 2.1.3. PEG-lipids can additionally balance the half-life and cellular uptake of LNPs [54]. For this reason, the PEG amount is today kept to a minimum of 1.5 mol% of the total lipid content, after Semple et al. reported a 5-fold potency improvement when decreasing PEG content from 10% to 1.5% [54]. Considering that PEG can also affect cell–nanoparticle interactions by preventing a close approach between the two and impeding endosomal escape by preventing fusion [77]; some studies suggest that causing PEG lipids to diffuse from the LNPs upon administration would allow for intracellular delivery promotion [78].

Despite the well-characterized and studied molar compositions, little is known about how changing the overall composition might affect the LNPs’ biodistribution. Apart from the above-described study where DOPE and DSPC LNP formulations were compared [71], another important study was recently published in *Nature* about selective organ targeting of LNPs [79]. In this study, the authors concluded that it is possible to include additional lipids to tune LNPs’ base composition, and thus modify their surface charge to target specific organs such as the spleen and lungs. To achieve this, they performed several experiments with different ionizable lipid LNPs (5A2-SC8, MC3 and C12-200) which they tuned with positive or anionic lipids. Results showed that increased amounts of DOTAP achieved an increase in protein expression in the spleen and lungs, and results were reproduced when other cationic lipids were used. Meanwhile, negative lipids drove the protein expression to the spleen. Furthermore, authors described the enhancement in liver delivery when supplementing established LNPs with more ionizable lipids [79].

Similarly, it was also described that mannosylation of LNPs may enhance the uptake of nanoparticles by antigen-presenting cells (APCs). By incorporating a mannose-cholesterol amine conjugate to LNPs, an enhanced in vitro uptake in dendritic cells was observed as compared to non-decorated LNPs, a fact that translates into a faster onset of the antibody response upon administration by both intradermal (i.d.) and intramuscular (i.m.) routes [80]. Authors further investigated how the length of this LNP-surface decoration may influence antibody response, observing that vaccine priming response was improved with a higher chain length [81]. 

#### 2.1.2. Manufacturing Processes for LNPs

The production of LNPs is based on the ethanol dilution technique first described in the early 2000s. This method consists of lipid dissolution in an ethanolic phase, followed by rapid mixing using a T tube mixer with an aqueous buffer solution containing the nucleic acid at acidic pH. As these two solutions are mixed, the ionizable lipid becomes positively charged and complexes with the negatively charged nucleic acids at the same time as the overall lipid solubility is reduced by the dilution of ethanol. This forms unstable LNPs in terms of size, which will then need pH adjustment to raise their value to physiological pH, normally achieved by dialysis processes [51,52]. The use of continuous microfluidics has revolutionized the bench-top formulation and manufacturing process of LNPs, allowing for the accurate formation of LNPs with defined sizes, by altering and optimizing the mentioned parameters as well as reducing manufacturing time [82]. This technique involves the mixture of the lipids with the aqueous phase using a micromixer, and is highly reproducible, scalable and robust [82,83]. Recently, manufacturing considerations for the production of LNPs using both cationic and ionizable lipids have been extensively reviewed in this article [84].

When considering bench-top formulations, important factors need to be considered to obtain LNPs with desired characteristics. In a recent study, Terada et al. investigated the effect of bench-top siRNA-LNP preparation parameters, i.e., total lipid content, flow rate ratio and total flow rate, by systematically comparing formulations using the design of experiments (DoE) platform, providing a statistically relevant study [85]. Conclusions were driven with respect to the lipid concentration and flow rate ratio, stating that these two factors might be critical for size dispersion when formulating and manufacturing LNPs. Furthermore, this study points to the fact that the presence of nucleic acids—in this case siRNA—dramatically impacts the LNPs behavior within the whole formulation process as compared to empty LNPs [85], which is also in line with the conclusions driven by Sebastiani et al. [61]. This issue will be further described in the next section.

#### 2.1.3. Disclosing the LNPs’ Structure

The structure of LNPs has not been fully deconvolved yet and is still a matter of debate. The two main structures proposed, which are explained in this section, can be pictured in Figure 3. The first studies on LNP structures have been conducted mainly for siRNA-loaded LNPs, and more recently, several studies have also reported the structural organization of mRNA-loaded LNPs.

Starting with siRNA LNPs, various studies have been carried out since Leung et al. first proposed that LNPs have an electron-dense core in which nucleic acid would be located inside inverted micelles [83]. According to this hypothesis, the initial association of the ionizable lipid with the nucleic acid would form such types of micelles, which in turn would associate with more empty micelles, altogether structuring the core. This core would then be coated with polar lipids, i.e., DSPC and PEG-lipids [83]. Later, other studies suggested that components would be located forming a layered structure, where the phospholipids and the PEG-lipids would be located at the surface, and the cholesterol and ionizable lipids would form the core of the nanoparticle, reporting differences in the structure depending on the cargo [86]. Subsequently, experiments using cryo-TEM and small-angle X-ray approaches suggested that LNPs would be formed upon fusion of smaller particles occurring after rapid mixing [87]. Later, the same authors performed experiments with larger cargoes such as pDNA, mRNA and gold nanoparticles [88]. Results obtained supported the initial idea that the formation of LNPs would happen through fusion of smaller particles in a pH-dependent manner after the rapid-mixing process, and that this fusion would be highly dependent on the amount of PEG-lipid accumulated in the surface. Thus, PEG content would dictate LNP size, with a higher PEG content leading to smaller LNPs [88]. Recently, the same group published a study hypothesizing that the entrapment of siRNA into LNPs occurs through inversion of preformed vesicles [89].

Studies performed with mRNA formulations firstly suggested that the mRNA would be located inside water cylinders surrounded by ionizable lipids [90]. In a later study using MC3 LNPs, authors concluded that the shell of the particle would indeed be enriched with DSPC, MC3 and cholesterol, the latter being mainly present here. The core would be composed of MC3, where it would be present in double the quantity as compared to the shell [61]. In the same study, LNPs and ApoE-interaction analysis concluded that this protein corona could mediate an irreversible rearrangement of components, affecting the LNPs’ fate and protein expression efficacy [61]. Furthermore, recent evidence showed that cholesterol analogues can also change the LNPs’ structure, by switching spherical particles to polyhedral [91]. 

Despite these recent advancements and hypothesis, more studies are still needed to better understand the structural organization of LNPs. In this regard, a study performed by Blakney et al. [92] aimed to describe how the association strategy of the nucleic acid to the LNPs would dictate the LNPs’ colloidal properties, in vitro and in vivo behavior. To achieve this, authors compared two different approaches: encapsulation and complexation (i.e., interior vs. exterior RNA localization). For this purpose, they used two different types of LNPs: LNPs incorporating a cationic lipid (cLNPs, DDA or DOTAP) or an ionizable lipid (iLNPs, C12-200). In terms of physicochemical characterization, results showed that saRNA localization did not seem to dictate colloidal properties, i.e., no difference was seen when comparing LNPs of the same composition but different saRNA localization. In regard to the transfection efficiency, authors showed that for iLNPs it was higher when the RNA was encapsulated, as opposed to cLNPs, where higher transfection rates were achieved when the RNA was complexed [92]. This study is important as it gives an alternative approach to the paradigm of encapsulation RNA for efficient delivery. The possibility of complexing the saRNA onto the LNP surface could have the potential advantage of having a ready-to-use blank formulation for association of RNA when needed, allowing shorter RNA working times, easy updating of the sequence, and so on. 

#### 2.1.4. Stability Considerations

Undoubtedly, one of the biggest challenges when it comes to mRNA formulation is the stability of the biomolecule. As mentioned before, mRNA is extremely unstable and needs to be stored at −80 °C. The importance of stability lies in the fact that even a small change in structure can stop mRNA translation into proteins. Physical instability comprises denaturation and the loss of secondary structure, which is related to aggregation and precipitation and linked to poor translation efficiency [49]. However, according to Pogocki et al., the main stability issue of mRNA is chemical degradation, which occurs via oxidation and hydrolysis via the phosphodiester bonds that form the backbone of the molecule. In vivo, there is a major challenge due to the presence of RNA-ases, converting protection of mRNA into a key issue to guarantee its efficacy [8].

From a pharmaceutical perspective, achieving a formulation that can be stored at higher temperatures while preserving biomolecular integrity is in fact one of the biggest challenges. To this end, studies evaluating pH, temperature and lyophilization techniques had been previously conducted with siRNA-containing LNPs, showing that when stored in aqueous media, refrigeration kept LNPs stable for the longest time, as compared to −20 °C or room temperature (RT). Interestingly, pH seemed to not be a factor influencing stability. Remarkably, authors showed that stability was protected upon freeze-thaw cycles when cryoprotectants such as sucrose or trehalose [93]—two excipients used in the marketed vaccines—were used. In this regard, more recently, a novel class of mRNA reactivity that leads to loss of activity has been reported: the formation of mRNA-LNP adducts though the covalent union between some reactive lipids and the nucleobase [24,94]. It is also worth noting that mRNA could potentially have more stability issues than siRNA, as it is a longer and more complex molecule. Indeed, Onpattro^®^ siRNA-LNP formulation has a shelf life of three years at 4 °C [95], while the BioNTech/Pfizer vaccine is reported in the technical documents to be stable for just 6 months between −90 °C and −60 °C [24]. However, Pfizer and BioNTech have submitted data on increased stability, proposing that the vaccine can be stored at −25 °C to −15 °C, and later in refrigerator storage, for up to one month once unfrozen. On the other hand, the Moderna vaccine can be stored frozen for up to 7 months and it is stable in refrigeration for up to 3 months [23].

### 2.2. Additional Lipid-Based Delivery Vehicles for mRNA

As introduced in Section 2.1, LNPs are today the most-researched delivery vehicle for mRNA delivery. Nevertheless, LNPs are not the only vehicle under investigation, and it is worth noting the importance of others. Both lipids and polymer materials hold tremendous potential, especially because it seems apparent that the idea of having one delivery system that fits all kinds of nucleic acids is no longer accepted. As such, polymeric nanoparticles and materials such as polyethyleneimine, polyacrylates, polyesters and polyamines, among others, are currently evolving as an alternative for lipid carriers. Nonetheless, this review will be focusing only on the newest advances in lipidic nanoformulations, leaving aside polymeric materials as they have been extensively covered in recent reviews [96,97].

#### 2.2.1. Lipoplexes

It was in the 1970s when Dimitriadis et al. succeeded in efficiently delivering encoded rabbit globin mRNA to mouse spleen lymphocytes for the first time, using large unilamellar liposomes [98]. At the same time, Dray et al. published results on protein translation after treating HEp-2 cells with mRNA encapsulated in liposomes [99]. However, due to the physicochemical characteristics of nucleic acids, traditional approaches used for small molecule encapsulation in response to pH changes had not been successful [52]. As mentioned in Section 2.1, LNPs also evolved from liposomes; however, also using approaches relying on the use of complexing agents, different delivery vehicles emerged, and are typically disclosed as lipoplexes (LPX, Figure 3C).

Regarding the composition of LPX, the most commonly described materials for mRNA delivery include several lipids such as DOPE, DOPC, DSPC and cholesterol, as well as complexing agents such as cationic lipids such as DOTAP, DOTMA and DC-Cholesterol; ionizable lipids such as MC3 or C12-200; or other positively charged peptides or polymers. Formulation methods described in the literature include the traditional approach of hydration lipid film [100], pre-complexation of the cationic component with the nucleic acid [101,102] and the simple mixture of lipids dissolved in ethanol with an aqueous phase containing the nucleic acid [103,104].

As mentioned, the use of complexing agents to link the mRNA to the nanocarrier is a commonly used strategy and normally involves cationic lipids. For instance, Sahin et al. showed in 2018 that an intravenously (i.v.) administered DOTMA/DOPE LPX formulation was capable of triggering INF-γ and T-cell memory upon administration of antigen-encoding mRNA [105]. On the same note, DC-Cholesterol/DOPE LPX have been studied for α-1-antitrypsin-encoding mRNA-protein production, showing minimal animal toxicity [106]. Van Hoecke et al. also used cationic LPX to study how the route of administration could impact T-cell activation [107]. More recently, Salomon et al. carried out a study with LPX containing DOTMA and DOPE, showing the potential of a CD4 neoantigen vaccine to increase the antitumor activity of local radiotherapy in mice [108]. Regarding SARS-CoV-2, a vaccine investigation using LPX has also been carried out. In a very recent study [109], the potential approach of using cholesterol/DOTAP LPX was evaluated preclinically resulting in a 30 µg mRNA dose, achieving immune activation after three doses. Interestingly, some studies have also been conducted for cationic LPX for other routes of administration other than i.v. and i.m. Interestingly, Dhaliwal et al. have shown the potential of DPPC/DOTAP/cholesterol LPX for mRNA delivery to the brain via intranasal (i.n.) administration. Results showed dose-dependent and region-specific distribution of mRNA when LPX are used, as opposed to naked mRNA administration [110]. 

Despite the fact that cationic lipid usage is still a main approach when formulating LPX, another approach has been described by Zhang et al. [102]. This strategy involves the use of a positively charged polymer, i.e., protamine, which complexes the nucleic acid prior to the formation of the nanoparticle. In this study, they succeeded in efficiently delivering T34A mRNA locally and systemically, improving the results on their DNA counterparts and also with a better safety profile on colon-cancer gene therapy [102]. The same team also proposed the use of this delivery vehicle for immunotherapy when they succeeded in delivering IL-15 cytokine mRNA in vitro and in vivo [101]. Later on, they also reported the use of this strategy to efficiently deliver a neoantigen mRNA vaccine using a positively charged peptide as complexing agent, i.e., DP-7 [111]. More recently, LPXs using KALA peptide as a complexing agent have also been reported [100] further evidencing the fact that other complexing agents can be used.

An interesting study regarding the complexing lipid type and localization aimed to characterize how this component affects the cellular uptake and mRNA expression in different cell lines in vitro and after intradermal (i.d.) administration. For this purpose, they used LPX composed of cholesterol, DOPE and different ionizable (C12-200, MC3), cationic (DDA, DOTAP) and zwitterionic (cephalin) lipids. The most relevant conclusion was that the resulting overall LPX-surface charge was the most important predictive factor for efficient protein expression [112].

In addition to complexing-agent modification, tuning the composition of LPX has also been studied. For instance, Ziller et al. published results on how EPC/DOPC/DOTAP LPX could be further modified to achieve controlled-release effects [104]. Furthermore, similarly to that which Goswami et al. [80,81] studied for LNPs, and as previously commented in Section 2.1.1, the development of mannosylation strategies through the incorporation of mannose-modified lipids has also been proposed for LPX for specific targeting to macrophages, achieving positive results in the induction of a stimulatory immune response upon mice vaccination [113,114]. 

#### 2.2.2. Cationic Nanoemulsions

Another form of lipid nanoformulations that are currently under preclinical and clinical investigation are cationic nanoemulsions (NEs), represented in Figure 3D. These delivery vehicles have been extensively used in nanomedicine for different kinds of drugs and applications thanks to their versatility and ability to encapsulate different types of therapeutic molecules [115,116,117] Emulsions are by definition a mixture of two immiscible phases that are stabilized by an emulsifier or surfactant, normally added to the continuous or external phase to stabilize the internal phase. Depending on the nature of the phases, emulsions can be categorized as oil-in-water (O/W) or water-in-oil (W/O) emulsions. Furthermore, emulsions can be classified depending on their droplet size as coarse emulsions or nanoemulsions, and there has in fact been some debate on nomenclature when considering nanoemulsions or microemulsions [118]. In this term, nanoemulsions are exclusively formed by spherical nanosized oil droplets; however, they are not thermodynamically stable, as microemulsions are [119]. Although microemulsions can exist in the nanosized ranges [119], we will refer to nanoemulsions here when considering only kinetically nanosized emulsions.

When considering formulation methods, NEs can be obtained by different methods, such as high-pressure homogenization, microfluidization or sonication. The technique employed in the manufacturing process and the type of lipids and formulation parameters have a huge impact on the physicochemical properties of the final formulations [115,117]. Undoubtedly, the simplest approach to formulate these nanoemulsions is the ethanol-injection method [120].

Specifically for gene-delivery purposes, NEs usually contain a hydrophobic oily core surrounded by phospholipids and other surfactants, and similar to what was proposed for LPX, cationic NEs typically contain a cationic component which complexes the nucleic acid. On this behalf, recent modeling of a siRNA-loaded nanoemulsion has been performed, finding it to have a compact shape in which the hydrophobic domains of the phospholipids and the oil are placed in the core, while the polar groups form a hydrophilic shield on the exterior of the particle. Moreover, ethanol molecules would be placed throughout the system, not contributing to the nanoemulsion’s size [121]. The siRNA would be attached to the nanoemulsion via non-covalent interactions with the cationic lipid. Interestingly, the physical radius of the nanoemulsion did not increase after the addition of the siRNA [121]. Apart from siRNA [122], NEs have been proposed for pDNA [123,124,125], miRNA [126] and—of the interest of this review—saRNA delivery [127].

Major advancements when considering preclinical and clinical development for cationic nanoemulsion technology have been achieved with saRNA [127,128]. The same formulation, composed of Tween80, Span 85, DOTAP and squalene, which initially showed prolonged levels of saRNA protein expression [127], was chosen to be preclinically tested in different animal models and for different applications. Notably, this formulation provided the first evidence in a non-human primate species that HIV vaccination with saRNA technology is safe and immunogenic [129]. Later, Brazzoli et al. studied the potential application of this formulation against the influenza virus, demonstrating good immunogenicity in ferrets and mice, exhibiting both T-cell and antibody responses [130]. Furthermore, it was also investigated for the Venezuelan equine encephalitis virus (VEEV) [131] and rabies virus [132]. A recent study described the development of a Zika virus vaccine using this same technology, and when comparing two different vaccine candidates, both elicited potent neutralizing antibody responses in mice and non-human primates [133]. Today, there is one cationic nanoemulsion in clinical trials for rabies virus (NCT04062669), as can be seen in Table 1.

As the reader can acknowledge by the information given so far, several delivery strategies are being proposed for RNA delivery. However, until recently, no study was conducted to make a head-to-head comparison of different formulations. A recently published study by Blakney et al. evaluated how the delivery vehicle might impact saRNA protein expression and vaccine immunogenicity [112]. To achieve this, authors compared pABOL, a polyplex formulation, which has previously shown its potential in saRNA delivery [134], with several LNP compositions, varying ionizable lipids and phospholipids (DOPE vs. DSPC). Formulation characterization results showed no major differences between colloidal properties and encapsulation efficiency, irrespective of their composition. The main difference was the surface charge; positive for pABOL and neutral for LNPs. However, when in vivo studies were performed, results showed an increased protein expression upon i.m. administration with the pABOL formulation. Within LNP formulations, DOPE-containing nanoparticles showed higher protein expression. Interestingly, when vaccine immunogenicity was investigated, LNPs exhibited higher IgG titers after the second shot, despite pABOL exhibiting higher protein expression levels upon i.m. administration. Higher antibody titers were also translated to higher protection against influenza virus with LNPs. When routes of administration were compared (i.m. vs. i.n.), i.m. administration of 1 ng of saRNA LNPs exhibited similar antibody titers to 1 µg of saRNA formulated in pABOL nanoparticles. An important factor describing vaccine immunogenicity is the capacity to achieve mucosal antibodies upon i.m. administration. In this study, mucosal antibodies were found in the majority of the doses given by LNPs, but not with pABOL. When given i.n., LNP formulations exhibited systemic antibody titers; however, these were lower than the same dose i.m. Overall, this study shows that the selection of the delivery vehicle can drive the fate and the therapeutic application and effect [112].

When considering the nanoparticle’s physicochemical properties, surface charge has been previously reported to be involved in the nanoparticle’s biodistribution [106], as well as the phospholipid selection [105]. Furthermore, the fact that LNPs exhibited a higher immunogenicity can be explained by increasing evidence that LNPs can act as adjuvants [135]. In this study, Alameh et al. compared three different treatments: first, rHA mRNA-loaded LNPs; second, empty LNPs (e-LNPs) with hemagglutinin recombinant protein (rHA); and third, a well-known adjuvant (Addavax) with rHA. Results showed that the first and second groups exhibited similar antibody titers, demonstrating the adjuvant capacity of e-LNPs [135]. 

### 2.3. Additional COVID-19 Vaccines Based on mRNA and Delivery Vehicles

Apart from the Moderna and BioNTech/Pfizer mRNA approved vaccines based on LNPs, it is worth mentioning that other companies are developing additional mRNA vaccines worldwide. That which has caused the most expectation so far is the vaccine from CureVac, named CVnCov, which was also expected to be stable at 4 °C, making it available in low-income countries; a very relevant and potential advantage over the Moderna and BioNTech vaccines. However, clinical data were not as good as expected. Results of their 40,000-participant clinical trial (NCT04652102) unexpectedly resulted in only 47% efficacy, as opposed to over 90% efficacy reported by the approved vaccines [136]. Several hypotheses have been described, such as the presence of new SARS-CoV-2 variants in the countries in which the study was held. However, Moderna and BioNTech/Pfizer (as well as different-technology vaccines) have shown to be efficacious against the new variants, including the Delta variant, which was first described in India [33,34,35]. In terms of delivery, to the best of our knowledge, CureVac LNPs have a similar composition to the BioNTech/Pfizer vaccine, but contain a lower mRNA dose, only 12 µg [49]. At the beginning of the clinical studies, Kremsner et al. compared different dosage regimens for CVnCoV and found that higher doses triggered too many side effects [137]. The level of neutralizing antibodies found in participants dosed with 12 µg was comparable to people who had had the infection; however, it was lower than recipients of the Moderna and BioNTech/Pfizer vaccine [137]. Of an important note, the CureVac vaccine uses unmodified mRNA, without introducing chemical modifications that can be fundamental for its efficacy and safety, as commented in Section 1.1. It is soon to conclude the reasons behind these results; however, CureVac’s clinical trial is still ongoing. CureVac has announced their work in optimizing these results as well as updating the vaccine for the new variants [138]. 

Also using LNPs, Translate Bio and Sanofi are developing MRT5500, currently under phase 1/2 (NCT04798027) clinical trials. In vivo preclinical results showed that it produces a strong immune response [139]. Other mRNA vaccine candidates refer to ARCT-021, from Arcturus Therapeutics, currently under phase 2 clinical trials. This vaccine contains saRNA encapsulated into a lipid-based LUNAR^®^ patented delivery system—the structure and composition of which are not disclosed—and it has shown to produce an immune response similar to the one produced by the people who are infected (NCT04668339). On a similar note, Gennova Biopharmaceuticals and HDT Bio are also working on a saRNA vaccine, which links the RNA in the exterior of ‘LIONS’ (lipid inorganic nanoparticles) and is currently under phase 1/2 after showing promising results in preclinical models [140]. Other companies and research institutes such as Daiichi Sankyo, Elixirgen Therapeutics (NCT04863131), Walvax Therapeutics (NCT04847102) Chulalongkorn University (NCT04566276), Providence Therapeutics (NCT04765436) and Stemirna (ChiCTR2100045984) are also working on the development of more efficacious and safe mRNA vaccines, with nondisclosed formulations.

## 3. Biomedical Applications of mRNA Using Nanomedicine

**Figure 4:**
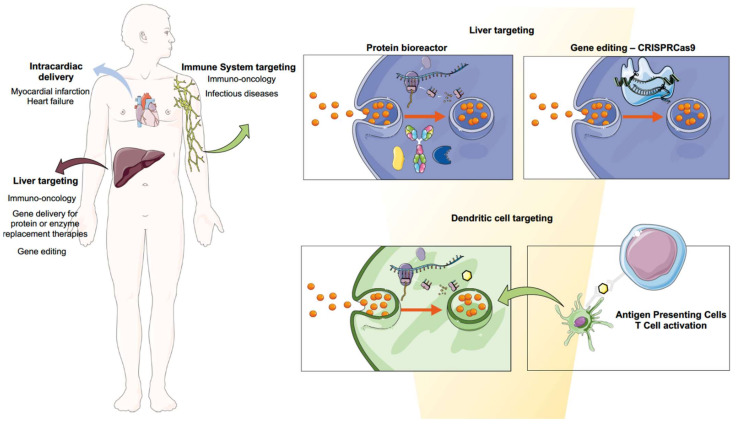
Biomedical applications for mRNA therapeutics. Immuno-oncology treatments can be envisioned using the liver as a factory of therapeutic proteins such as neoantigens or antibodies, as well as by using the antigen-presenting cells’ physiological function to induce immune activation towards cancerous cells. The latter can also be applied for the development of safe and efficacious mRNA and saRNA vaccines against infectious diseases. Gene delivery for therapeutic protein synthesis or restoration of a particular enzymatic function as well as gene editing using CRISPR cas9 technology can be achieved by targeting the liver. Cardiovascular regeneration can be achieved by intracardial delivery of mRNA encoding for growth factors.

The approval of mRNA vaccines using LNPs can indeed be considered the biggest milestone to foster the clinical translation of many other mRNA nanoformulations that can address current clinical needs. Indeed, the technology can be applied to a broad spectrum of applications and therapeutic areas. Vaccines for infectious diseases might be the first application that has reached the market, but nanoformulated mRNA has the full potential to answer clinical demands related to the needs of therapeutic proteins. In the next sections, we aim to provide a full overview of the most promising developments to date. Biomedical applications for mRNA therapeutics are pictured in Figure 4.

### 3.1. Infectious Diseases

Despite the fact that COVID-19 vaccines have been the first marketed mRNA formulations, this technology has been researched for decades using different delivery approaches, from LPX to cationic nanoemulsions to polymeric nanoparticles [9]. Since mRNA loaded into liposomes demonstrated for the first time that it could trigger a specific T-cell response against influenza virus in mice [141], the field has enormously advanced. In fact, only in 2019, 15 mRNA candidates entered clinical trials [10]. Because this area has very recently been covered in an extensive review [142], we will only showcase some of the most promising approaches, redirecting the reader to this article for further information. Nonetheless, all the clinical trials for infectious diseases other than COVID-19 are shown in Table 1. In this regard, clinically, respiratory syncytial virus (RSV) and influenza virus are amongst the most advanced clinical candidates.

Furthermore, preclinically, one of the most important applications is the HIV vaccine. HIV currently affects 38 million people; these numbers are expected to increase by 2030 [143]. This virus has been extensively researched for decades; however, its retroviral nature, along with the fact that it possesses a very high antigen diversity in its envelope, make it very difficult to achieve good outcomes [144]. In recent years, several studies have tried to deliver mRNA-vaccine encoding for HIV proteins; however, their success has not been as expected [127,129,145]. Nevertheless, a recently published study has shown broad neutralization and reduction in the risk of infection in rhesus macaques upon the administration of mRNA encoding for both HIV-1 envelope protein and simian immunodeficiency virus Gag proteins, which would be responsible for forming virus-like particles (VLP) [146]. A different approach based on targeting hepatocytes, which will be further discussed for immuno-oncology applications in Section 3.2.2, has achieved good results. This study involved the delivery of mRNA encoding the light and heavy chains of VRC01, a neutralizing antibody against HIV-1. Using LNPs, they were able to achieve robust protein expression in the liver. Furthermore, prophylactic immunization was also achieved at a dose of 0.7 mg/kg as opposed to the 10–20 mg/kg needed if the recombinant protein is administered [147]. 

Another important preclinical advancement was achieved with the Ebola vaccine. Ebola virus (EbolV) caused the deaths of more than 11,000 lives across West Africa from 2014 to 2016 [148]. This lethal virus causes a hemorrhagic fever that is fatal in up to 50% of the patients [148]. In 2019, one viral vector-based Ebola vaccine was approved by the FDA; however, clinical trials reported some safety concerns. As explained in the introduction, from this point of view, mRNA vaccination presents an advantage. In a study on guinea pigs, 20 μg of glycoprotein-encoding mRNA was administered using LNPs as a delivery vehicle, resulting in good antibody titers and protection from death [149].

Lastly, it is important to note the advancements achieved in other infectious diseases other than viruses. In this regard, *Plasmodium*, the causal agent of Malaria, is highlighted next. Very recently, the first malaria vaccine was approved by WHO [150]. The RTS, S malaria vaccine is a recombinant protein vaccine which has shown to prevent lethal disease, and is especially efficacious when given together with pharmacological treatment [150]. Several studies have also been conducted towards the development of an mRNA vaccine against malaria. The first is an saRNA-based [151] encoding for *Plasmodium*-secreted cytokine macrophage migrating inhibitory factor, which had previously shown efficacy [152]. In this study, anti-*Plasmodium* antibodies and protective T-cell memory were achieved [151]. In another study, *Plasmodium falciparum* acid-rich protein (PfGARP) mRNA-encoded LNPs were dosed to infected aotus monkeys, showing reduced levels of the parasite after three doses [153]. 

Altogether, these advancements provide strong evidence of the possibilities ahead regarding mRNA vaccinology, which seems to provide necessary advantages as the next vaccine technology, especially when considering future pandemics. 

### 3.2. Immuno-Oncology

RNAs are critical for gene expression in their multiple forms, whether in non-coding forms such as miRNA, siRNA or tRNAs that regulate transcription and translation, or in coding forms such as mRNA [154]. Cancer immunotherapy has been a game changer since the approval of the immune checkpoint inhibitor ipilimumab in 2011 [155]. The number of immuno-oncology (IO) drugs in the development pipeline in 2020 grew by 22% compared to 2019, accentuating the high interest in these therapies. Immunomodulators, cell therapies and cancer vaccines represent the most important trends [156]. In fact, these therapies are often given simultaneously, with one example of this being TriMiX, whose composition is CD70, CD40 ligand and TLR4 [157]. However, scientists have met a few challenges when it comes to the new development of these immunotherapeutic agents, mostly related to off-target toxicity [158]. Thus, mRNA technology can offer advantages in terms of scalability, cost and production issues, widening immunotherapy use worldwide. Altogether, immunotherapy combined with nanomedicine could bring a potential solution: to engineer the delivery of immunomodulators, focusing their action on target tissues such as tumors or tumor-draining lymph nodes, or specific cell types, so as to control the timing and location of immunomodulation [158]. To achieve this, two main strategies can be performed: the first, by targeting DCs and taking advantage of their natural immune role; and the second, by making use of liver capacity to synthesize proteins. In the following sections we will describe both mechanisms in depth.

#### 3.2.1. Cancer Vaccines: Targeting Dendritic Cells

The most well-known strategy for treating cancer with mRNA is cancer vaccines, in which mRNA is transcribed into a protein which in turn trains the immune system to fight against the tumor. Thus, rather than being prophylactic, cancer vaccines seek to stimulate cell-mediated responses. Therapeutically, this technology can be applied (1) by using shared antigens for the same tumor to stimulate immune activation, or (2) by designing a personalized cancer vaccine which possesses the individual’s neoantigens, i.e., proteins preferentially expressed in cancerous cells but not in the patients’ healthy tissue, such as growth-associated factors or antigens [9].

**Figure 5:**
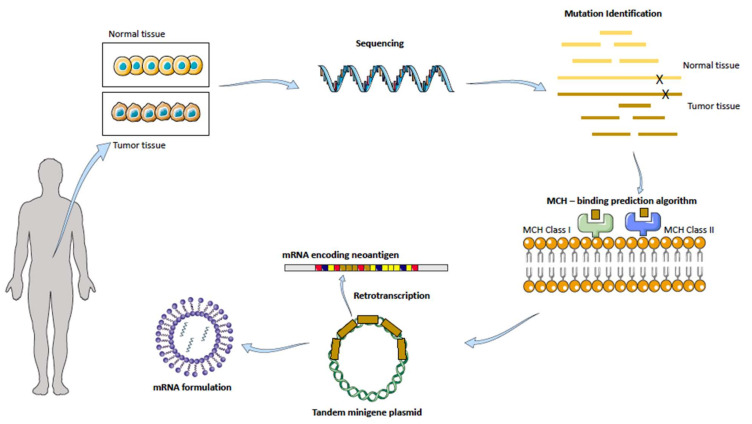
Personalized mRNA cancer vaccines. Healthy and tumor tissue are isolated from the patient sample, analyzed and compared in order to find mutations present in the tumor tissue. Bioinformatic tools are used to predict the MCH Class I and II binding. A tandem gene encoding for several neoantigens is then designed and retrotranscribed to mRNA. Finally, by making use of nanotechnology, the mRNA is formulated and delivered. Adapted with permission from [159], published by Nature Reviews Drug Discovery, 2018.

The way the latter strategy (2) is approached involves the most advanced tissue gene sequencing: a patients’ tumor and healthy tissue samples are taken and analyzed looking for tumor-associated antigens, as pictured in Figure 5. 

The technology relies on a special algorithm prediction of binding strength to self-major histocompatibility complex (MHC) receptors. After mutation identification, neoantigens are ranked based on how well they would bind to this receptor, which will then drive the activation of T cells, followed by the immunological response against that specific neoantigen. Upon administration of the mRNA into a suitable, stable and efficient formulation, DCs are transfected, and due to their antigen-presenting cells’ nature, they localize the neoantigen onto their MHC receptor located in their surface, which will be then presented to the T cells, starting the immune cascade [159,160]. 

Today, both strategies described, (1) and (2), are under clinical evaluation in phase 1 or 2. Melanoma, prostate cancer, triple-negative breast cancer and head and neck cancer are some of the biomedical applications in which this technology is being clinically investigated (Table 2). An extensive review on this matter has been recently published [161].

#### 3.2.2. The Liver as a Factory of Immunomodulatory Proteins

As commented in the previous section, targeting DCs is the main strategy towards the development of cancer vaccines. Nonetheless, this is not the only approach in which mRNA can be used for immunotherapy. This approach is based on the on the fact that nanoparticles have the intrinsic capacity to accumulate in the liver [162], which has been for many years the core center of the research. Thus, targeting the liver can be achieved by exploiting its capacity for protein production in order to build up an endogenous factory of therapeutic proteins, which will then be released into systemic circulation. In fact, the feasibility of targeting the liver for mRNA delivery for the endogenous production of immunomodulators can be envisioned by considering advances in other research areas, such as protein replacement therapies with mRNA, which will also be covered in Section 3.3. Furthermore, this strategy also overcomes the main disadvantages of many immunomodulator administrations, such as their short half-life and subsequent long perfusions and recurrent visits to the medical doctor’s office, as well as their high production cost [163]. 

This mRNA application has been studied for two types of recombinant proteins: immunomodulators and antibodies. The production of immunomodulators, such as cytokines, has already been proven to be an achievable strategy. For instance, intratumoral administration of LNPs with mRNA-encoding cytokines IL-23 and IL-36 and the T-cell costimulatory OX40L was proven to lead to tumor regression in colon cancer and melanoma models and is now under clinical trials (NCT03739931) [164]. In another study using IL-12, a well-known cytokine [165], Lai et al. reported mRNA-encoding IL-12 LNPs to be a good candidate for slowing down the progression of MYC oncogene-driven human hepatocellular carcinoma, with good biodistribution within the tumor and no toxicity. Treatment reduced liver-tumor burden and increased survival in MYC-induced HCC transgenic mice [166].

Interestingly, co-delivery of cytokines and antibodies has proven to have potential for treating different types of cancer [166,167,168]. In fact, the use of mRNA to endogenously produce antibodies has also been achieved. Apart from the already-commented HIV study in Section 3.1 [147], another study with trastuzumab aimed to apply this strategy to tackle oncology. Rybakova et al. succeeded in efficiently delivering mRNA encoding for trastuzumab, a monoclonal antibody widely used for breast cancer. They found a dose-dependent increase in trastuzumab protein levels in mouse serum after 24 h administration. When the administration of the monoclonal antibody Herceptin^®^ was compared, results revealed similar half-lives for both antibodies (i.e., endogenously produced and Herceptin); however, the mRNA-based delivery resulted in a 64% higher antibody serum concentration over the course of 30 days. Overall, a single dose of LNPs with the mRNA at 2 mg/kg showed a favorable pharmacokinetic profile when compared to a single injection of Herceptin at 8 mg/kg [169]. This technology has also been proven to be efficient for the delivery of bispecific antibodies [170]. Stadler et al. succeeded in achieving a promising therapeutic outcome by generating his-tagged bispecific antibodies directed against the T-cell receptor-associated molecule CD3, a tumor-associated antigen [170]. 

#### 3.2.3. CAR-T Cells

On a last note on immunotherapy, the CAR-T-cell field can also take advantage of mRNA technology. Chimeric antigen receptor (CAR) T-cell therapy is considered by many the next promise of cancer immunotherapy. In August 2017, the FDA approved the first CAR-T treatment, Kymriah^®^ (tisagenlecleucel), for the treatment of certain patients, both pediatric and young adults with B-cell acute lymphoblastic leukemia (B-ALL). Several months after that, in October 2017, the FDA approved Yescarta^®^ (axicabtagene ciloleucel) for adult patients with certain types of refractory B-cell non-Hodgkin lymphoma (B-NHL) [171] This approach consists of engineering lymphocyte T surfaces with tumor specific antigens that would bind to tumor-specific receptors. Right now, CD19-targeting CAR-T-cell therapy is approved for both B-cell acute lymphoblastic leukemia (B-ALL) as well as non-Hodgkin lymphoma (NHL), where it has demonstrated up to 90% complete remission when rayed in chemo-refractory B-cell malignancies [171]. Despite the promising positive outcomes on liquid tumors, CAR-T therapy has challenges in its application to solid tumors, including limited cancer-specific targets and non-persistence of the transferred CAR-T cells, allowing for low side effects but efficient tumor eradication [172], and research needs to be carried out to tackle solid tumors such as breast, melanoma or ovarian. With regard to solid tumors, in a recent paper published, T-cells were bioengineered with nanoparticle-encapsulating mRNA encoding for a single-chain variable fragment (scFv) that can specifically bind to CLDN6, an oncofetal cell-surface antigen for CAR-T-cell targeting. Results demonstrated that RNA nanoparticles can efficiently stimulate CAR-T cells [173]. 

### 3.3. RNA-Based Protein Replacement Therapies

As mentioned before, attempts to deliver therapeutic antibodies as mRNA molecules have been carried out with success with the aim of overcoming the drawbacks of administering the therapeutic protein. So far, protein replacement therapies are mainly focused on secreted proteins or proteins of bacterial origin. It is important to understand the processes following the translation of mRNA into proteins, which include folding, post-translational modification, aggregation into secretory granules and transport outside the cell. One of the most important things to consider are the signal peptides, as they take a great role in directing the protein secretion. Unless the signal sequence is optimized, extracellular mRNA should be transfected ideally into cells that the encoded protein is naturally secreted in [174]. A table with current clinical trials on going is provided in this section (Table 3).

#### 3.3.1. Cystic Fibrosis

Cystic fibrosis (CF) is a genetic disease which affects over 90,000 individuals all over the world. The F508del is the most prevalent CF-causing mutation, and affects approximately 82% of the CF population. This mutation leads to CFTR-protein misfolding, which is then arrested by the cell machinery and targeted to the proteasome, leading to its premature degradation [175]. Thus, mRNA-mediated replacement therapy can be applied to produce copies of the cystic fibrosis conductance receptor (CFTR), which is defective in CF patients. However, when it comes to delivery of mRNA nanoparticles to the lung, these nanoformulations not only need to be endocytosed, escape the endosome and release the cargo inside the cytoplasm, but they also need to migrate across viscous mucus, avoid clearance and uptake by the macrophages within this mucus and the airways and finally reach the pulmonary epithelial cells [175,176]. These additional biological barriers make the delivery of mRNA-loaded nanoparticles to the lung especially challenging. To date, the most advanced mRNA therapy for cystic fibrosis comes from Translate Bio and it is currently in Phase 1/2 (NCT03375047) [177]. 

#### 3.3.2. Rare Metabolic Diseases

An important area which would be enormously impacted by this technology are rare metabolic diseases. These disorders lack financial support, and thus, effective treatments. From this point of view, mRNA technology can entail an enormous advantage due to the cost-efficient matters already commented. Furthermore, the previous idea and background given on endogenously produced proteins can also be applied in this section.

##### Acute Intermittent Porphyria

Acute intermittent porphyria is a chronic disease caused by the hepatic deficiency of porphobilinogen deaminase (PBGD), the enzyme which catalyzes the transformation of porphobilinogen into protoporphyrinogen during the heme synthesis pathway [178]. This causes the accumulation of the porphyrin precursors γ-aminolevulinic acid (ALA) and porphobilinogen (PBG), which entails acute neurovisceral attacks associated with a high production of potentially neurotoxic porphyrin precursors [178]. Treatments so far are composed of hemin replacement therapy, which restores the regulatory heme pool in the liver and suppresses the accumulation of precursors; however, these reductions occur days after the injection and the levels of enzymes should be monitored closely. Although this is an efficacious treatment, 5% of patients suffer recurrent attacks over the years. Prophylactic hemin treatment is being more commonly used for chronic symptoms; however, repeated administrations have been shown to have important side effects [179] RNA therapies seem to give an answer to the treatment of this disease. The siRNA drug Givosiran^®^ aims to silence the ALAS1 enzyme, stopping the production of ALA and PBG. mRNA therapies are still in preclinical development. In this regard, Jiang et al. evaluated, along with the Moderna, the potential treatment of this disease with mRNA-encoding PBGD enzyme into LNPs in two models of acute hepatic porphyria. Furthermore, they also performed a multiple-dose study on non-human primates to further prove its translatability to humans. The results showed that administration of hPBGD mRNA led to expression of the therapeutic protein after 2 h post-administration, and most importantly, maintained its activity throughout the entire duration of the attack [180]. Overall, this study reported sustained efficacy and tolerability of the hPBGD mRNA after single and repeated administrations in mice, rabbit and non-human primates [180], which further evidenced the potential of mRNA for the treatment of this disease. 

##### Propionic Acidaemia (PA)

Propionic Acidaemia (PA) is a rare pediatric disorder caused by propionyl-CoA carboxylase (PCC) deficiency, which results in impaired propionate metabolism and accumulation of toxic metabolites, leading to a life-threatening condition. There are two disease subtypes, PCCA deficiency and PCCB deficiency, each of them referring to the different subunits forming PCC, respectively A (of 72 kDa) or B (of 54 kDa). Current treatments for this disease include fluid therapies or actions to remove the excess of toxic metabolites, but liver transplant is also a potential outcome for these patients. In this term, mRNA-therapeutic encoding for PCC enzymes can provide a solution. In this study, Jiang L. et al. report the use of two mRNAs encoding both human PCCA and PCCB. Results showed the restoration of ammonia levels and the function of the PCC enzyme in the liver; reducing toxic metabolites associated with this condition up to 6 months upon repeated administrations in mice [181]. Currently, mRNA-3927 from Moderna Therapeutics is under clinical trials (NCT04159103). 

##### Fabry Disease

Fabry Disease is an X-linked lysosomal storage disorder which results from the mutation of the gene GLA encoding for the lysosomal enzyme α-galactosidase A, responsible for glycolipids’ metabolism. Its loss of activity leads to fat accumulation within the lysosomes of multiple tissues, which in turn leads to clinical disease with fatal renal and cardiac function alteration [182]. Current treatments for this metabolic disease include enzyme replacement therapies named Replagal^®^ and Fabrazyme^®^ [183]; and migalastat [184], a small molecule chaperone. While these treatments have shown to slow the progression of the disease, the variability of response, immunogenicity and difficulties with the manufacturing of these therapies are needs which have not yet been met. In this context, the main studies with mRNA have been published so far, showing some advancements. Using mRNA encoding for α-galactosidase, DeRosa et al. demonstrated it is possible to achieve sustained delivery in a Fabry model mouse and in a non-human primate with a resulting reduction of relevant biomarkers [185]. Upon delivery, serum protein levels reached as high as 1330-fold over normal physiological values. Another study conducted by Moderna also showed promising results upon intravenous administration of LNPs, with a dose-dependent protein activity and substrate reduction maintained for up to 6 weeks in mice. Upon repeated administration, the pharmacodynamic response was sustained and the administration to non-human primates confirmed safety and translatability [186].

##### Methylmalonic Acidemia (MMA)

Methylmalonic Acidemia (MMA) is a rare metabolic disease which is characterized by mutations in the gene coding of the vitamin B12-dependent enzyme methyl-malonyl-CoA (MUT), causing partial or complete loss of activity. This enzyme catalyzes important metabolic reactions linked to the Krebs cycle, playing a central role in intermediary metabolism. Its partial or total lack of activity causes the accumulation of toxic metabolites and the alteration of mitochondrial oxidative phosphorylation. Clinically, patients affected with this disorder experience multisystemic complications which are more common in organs with high metabolic demand, such as the brain or heart [187].

There is currently no effective treatment for these patients, and this disorder is often treated by carnitine supplementation and protein-limited diets; however, mortality is still around 20%, and survivors can develop systemic complications. mRNA therapy encoding for human MUT using LNPs as delivery vehicles was studied by An et al. in two clinically relevant models. In both, a decrease in the enzyme substrate methylmalonic acid blood levels were achieved, with good pharmacokinetic properties. Importantly, none of the clinical models developed any hMUT antibodies upon repeated administration or hepatic inflammation [188].

##### Ornithine Transcarbamylase Deficiency (OTD)

Ornithine transcarbamylase deficiency (OTD) affects 6 in 100,000 people, and is the most frequent inborn error of the urea cycle. As other urea-cycle disorders, it results in ammonia accumulation which clinically translates to fatal neurological disorders. OTD is more specifically caused by a deficiency in ornithine transcarbamylase (OTC). Therapeutic options for these patients include a protein-restricted diet for life, with arginine and citrulline supplementation, but liver transplantation is the only therapeutic option in cases of severe OTC deficiency [189]. In this study, the mRNA administration resulted in increased OTC enzymatic activity up to 10 days after dosing, along with increased mice survival [190]. 

### 3.4. Gene Editing

Clustered regularly interspaced short palindromic repeat (CRISPR)-related systems have become the main tool when considering therapeutic approaches for the cure of genetic diseases. CRISPR-Cas9 complex is composed of two main elements: Cas9 protein, an endonuclease that is capable of DNA cleaving; and a single-guide RNA (sgRNA), which guides the Cas9 protein to the site of interest. This gene-editing tool is, therefore, based on the recognition of PAM (protospacer-adjacent motif) sequence and a complementary 20-nucleotide genomic sequence that induces DNA brakes, which are repaired by error-prone nonhomologous end-joining or precise homology-directed repair [191]. To date, CRISPR/Cas9-based systems are versatile and have extensively been used for biotechnological purposes such as knockout/knock-in animal models or cells. From a drug-delivery point of view, it is important to understand that intracellular delivery of both components is needed. Therefore, various in vivo studies addressing differing delivery approaches such as viral vectors, mRNA or recombinant protein have been performed. In this section we will focus solely on mRNA delivery, as it is the purpose of this review [191,192,193,194,195].

One of the approaches that have been most extensively researched are hepatic diseases. In 2016, a study on human hereditary tyrosinemia (HHT) showed slightly promising results on correcting the fumarylacetoacetate hydrolase (FAH) mutation. This was performed by the systemic delivery of Cas9 mRNA encapsulated in LNPs and sgRNA encapsulated in AAV particles. The final results demonstrated an overall gene correction of 6%, suggesting that this could be an effective approach for the treatment of a range of metabolic liver diseases. However, authors pointed out that one potential reason for the mild gene correction could be that the nanoformulations were not co-delivered, hypothesizing that the sgRNA suffered degradation during mRNA translation, and thus decreasing efficacy [195]. Following up on this, another study conducted by the same team aimed to deliver both Cas9 mRNA and sgRNA using LNPs for both components. In order to make it possible for the sgRNA to be efficiently delivered, they conducted several chemical modifications on the sequence, i.e., including the addition of 2’ hydroxyl (OH) groups on nucleotides, which resulted in highly improved results with an efficacy of 80% [196] This study is especially important as it highlights the importance of chemical modifications as well as the possibility of using synthetic nanoparticles, broadening up the industrial applications of gene editing. Similarly, Miller et al. reported successful results with a 95% protein knockdown in vitro, by making use of a zwitterionic lipid as a delivery vector by also co-delivering the different components [197] Adding to this latter study, Finn et al. reported a 97% reduction in serum protein levels upon the administration of Cas9 mRNA co-formulated with modified sgRNA into LNPs for gene editing of the mouse transthyretin gene in the liver [198]. To sum up, probably the most important advancement with regards to gene editing is the NTLA-2001 clinical study (NCT04601051), the first clinical trial so far with CRISPR-Cas9, conducted by Intellia Therapeutics for patients with hereditary ATTR amyloidosis suffering from polyneuropathy. This gene-editing medicine is based on the CRISPR Cas9 system and it uses mRNA-encoding Cas9 protein and the guide RNA encapsulated in a LNPs. Results showed mild adverse effects, and the administration led to a decrease in TTR after a single dose [199]. In October 2021, Intellia Therapeutics announced the beginning of a new CRISPR clinical trial, NTLA-2002, for the treatment of hereditary angioedema (HE), a rare autosomal dominant disorder that causes unexpected inflammatory attacks [200]. Altogether, these results present an undeniable precedent for gene editing. A current landscape on CRISPR-Cas9 clinical trials is provided next (Table 4).

### 3.5. Autoimmune Diseases

Autoimmune diseases are chronic and debilitating conditions which are caused by immune response against self-antigens. Disorders such as multiple sclerosis or diabetes type I affect millions of people worldwide. Current treatments available do not target the origin of the disease and are not curative. However, immune tolerance—defined as the state of unresponsiveness of the immune system (IS) to any substance that has the potential to trigger a response—has been described before as a potential therapeutic tool [201,202]. In this term, mRNA technology could potentially be used to induce self-tolerance, by administering a self-antigen encoded in mRNA. In a recent study, Krienke et al. [203] showed that it is possible to deliver myelin oligodendrocyte glycoprotein (MOG)-encoded mRNA by making use of the lipoplex-type nanocarrier to multiple-sclerosis mice models. Upon intravenous administration, modified mRNA was capable of triggering the proliferation of T-reg cells without inducing immune activation, preventing myelin damage and paralysis [203]. This is, to the best of our knowledge, the first proof of concept using mRNA technology for immune tolerance. 

### 3.6. Cardiovascular Diseases

Another potential for the development of mRNA therapeutics is treating myocardial infarction (MI) and heart failure (HF), the leading causes of death in many countries. MI leads to a massive loss of cardiomyocytes (CM), which are replaced by highly proliferating fibroblasts, resulting in loss of healthy tissue. These changes are irreversible, and along with oxidative stress and inflammation, the ultimate result is heart failure. Gene therapy with DNA-based or viral vectors has been used to try to induce cardiac regeneration, but has not had great success due to poor and uncontrolled gene expression. However, mRNA technology could give a plausible response for the treatment of ischemic injury, mainly by three different mechanisms: (1) inducing CM proliferation; (2) inhibiting heart cell death and attenuating inflammation; and (3) supporting cardiovascular regeneration. It is important to note that although mRNA applications in cardiology are progressing quickly, right now research is focused on intracardiac administration, as it is the most effective delivery method so far; however, it causes stress and local injury to the tissue. Therefore, it is crucial to develop a delivery vehicle that can target the heart and that can be then administered s.c. or i.v. [204,205]. mRNA encoding for vascular endothelial growth factor A (VEGF-A) is currently under clinical phase 2 (Table 5) in a collaboration between AstraZeneca and Moderna Therapeutics after showing the induction of cardiovascular regeneration in several studies. In addition, Magadum et al. were able to induce CM proliferation by using mRNA encoding for FSTL1 and Pkm2 [204].

## 4. Highlights and Conclusions

The development and commercialization of mRNA COVID-19 vaccines have pivoted the way towards future applications of mRNA medicines by finally finding a solution to the problem of delivery. Despite concerns from detractors about a lack of applications for nanotechnology, nanomedicine has now demonstrated that it is a translational, relevant and much-needed approach to engineer new genetic drugs. Thus, it is now clear that the full potential of mRNA therapeutics, along with its advantages could not be envisioned without nanomedicine. As presented in this review, mRNA therapeutics are advantageous over other gene therapies in many important ways, such as improved safety, ease of manufacture and interchangeable mRNA sequence.

However, despite the importance of having addressed the question of delivery, several limitations are still present when considering mRNA therapeutics and nanoparticle formulations. In terms of materials used for nanoparticles, there is still a need to find new biocompatible and—ideally—biodegradable compounds that would allow repeated and recurrent administration, which is needed for some of the applications reviewed in this article, such as protein replacement therapies. In this sense, a high-throughput screening of new lipids and polymers that can retain encapsulation efficiency is a high priority. Moreover, understanding, minimizing or even taking advantage of the pro-inflammatory response to LNPs is crucial for the future development of new mRNA-based drugs. Additionally, the COVID-19 pandemic has proven that new materials capable of maintaining mRNA stability at higher temperatures in order to achieve an easy-to-transport technology is crucial, especially considering equitable access to vaccines. 

In regard to the mechanism of action, assays to investigate endosomal escape and release of the cargo once endocytosed, as well as endosomal trafficking, should be considered as relevant assays to undertake during the preclinical development of mRNA medicines. This matter is of particular interest, as endosomal escape has not been fully understood yet, and is one of the main problems regarding the transfection efficiency of mRNA nanoparticles.

Considering biomedical applications, the restrained delivery to DCs and natural accumulation of nanoparticles in the liver might be one of the most challenging and important issues to tackle, as it averts the potential of this application to other organs. In this regard, recent studies on selective organ targeting [80] have shown that by correctly bioengineering nanoformulations it is possible to address this inconvenience. Recently, however, this mechanism was proposed based on the formation of the protein corona, by which nanoparticles interact with blood proteins, redirecting them towards different organs. In this way, a new paradigm—endogenous delivery—starts, in which the carriers’ composition is designed to interact with specific and known proteins to overcome the hepatic barrier [206].

Today, after millions of doses have been administered worldwide, the available data on mRNA COVID-19 vaccines showcase a great safety profile together with high real-world effectiveness, providing more evidence on how this technology can reshape the future of modern medicine for many therapeutic applications, from cancer vaccines to gene editing. 

## Figures and Tables

**Table 1:** mRNA Nanomedicines for Infectious Diseases in Clinical Trials. A list of clinical trials for infectious diseases other than COVID-19 is provided in this table.

Infectious Diseases Other than COVID-19					
Therapeutic	Clinical Application	Formulation	Phase	NCT Number	Sponsor
mRNA-1653	hMPV/PIV3	Modified mRNA-LNPs	Phase I	NCT04144348 NCT03392389	Moderna
mRNA-1345	RSV	Modified mRNA-LNPs	Phase I	NCT04528719	Moderna
mRNA-11777	RSV	Modified mRNA-LNPs	Phase I	Unregistered	Moderna, Merck
mRNA-1172	RSV	Modified mRNA-LNPs	Phase I	Unregistered	Moderna, Merck
mRNA-1893	Zika	Modified mRNA-LNPs	Phase I	NCT04064905	Moderna
mRNA-1325	Zika	Modified mRNA-LNPs	Phase I	NCT03014089	Moderna
mRNA-1647	CMV	Modified mRNA-LNPs	Phase II	NCT04232280	Moderna
mRNA-1647	CMV	Modified mRNA-LNPs	Phase II	NCT03382405	Moderna
mRNA-1443	CMV	Modified mRNA-LNPs	Phase I	NCT03382405	Moderna
MRT5400	Influenza A (H3N2)	Not disclosed	Phase I	Unregistered	TranslateBio, Sanofi
MRT5401	Influenza A (H3N2)	Not disclosed	Phase I	Unregistered	TranslateBio, Sanofi
mRNA-1010	Influenza A, Influenza B	Not disclosed	Phase II/III	NCT04956575	Moderna
mRNA-1851	Influenza A (H7N9)	Modified mRNA-LNPs	Phase I	NCT03345043	Moderna
mRNA-1440	Influenza A (H7N8)	Modified mRNA-LNPs	Phase I	NCT03076385	Moderna
mRNA-1388	Chikungunya	Modified mRNA-LNPs	Phase I	NCT03829384	Moderna
CV7201	Rabies	Non-modified mRNA- in RNActive	Phase I	NCT02241135	CureVac
CV7202	Rabies	Unmodified mRNA-LNPs	Phase I	NCT03713086	CureVac
GSK3903133A	Rabies	saRNA–cationic nanoemulsion	Phase I	NCT04062669	GSK

Abbreviations: hMPV/PIV3—human metapneumovirus/parainfluenzavirus type 3; RSV—respiratory syncytial virus; CMV—cytomegalovirus; HPV—human papillomavirus.

**Table 2:** mRNA Nanomedicines for Oncology in Clinical Trials. A list of clinical trials for oncology applications, including the subsections described above, is provided in this table.

**Oncology**					
**Cancer Vaccines**					
**Shared Tumor Antigens**					
BNT111	Advanced Melanoma	Modified mRNA-LPX	Phase II	NCT04526899	BioNTech
BNT112	Prostate Cancer	Modified mRNA-LPX	Phase I/II	NCT04382898	BioNTech
BNT113	HPV16+ head and neck cancer	Modified mRNA-LPX	Phase II	NCT04534205	BioNTech
BNT114	Triple-Negative Breast Cancer	Modified mRNA-LPX	Phase I	NCT03815058	BioNTech
BNT115	Ovarian Cancer	Modified mRNA-LPX	Phase I	NCT04163094	BioNTech
mRNA-5671	Carcinoma, NSCLC, Pancreatic and colorectal neoplasms	Non-disclosed	Phase I	NCT03948763	Merck/Moderna
**Personalized cancer vaccines**					
mRNA-4157	Melanoma	Modified mRNA-LNPs	Phase II	NCT03897881	Moderna
BNT122	Advanced Melanoma	Modified mRNA-LPX	Phase I	NCT03815058	BioNTech
mRNA-4157	Solid Tumors	Modified mRNA-LNPs	Phase I	NCT03313778	Moderna
mRNA-2752	Solid Tumors	Modified mRNA-LNPs	Phase I	NCT03739931	Moderna
CV8102	Solid Tumors	Not disclosed	Phase I	NCT03291002	CureVac
V941	Solid Tumors	Not disclosed	Phase I	NCT03948763	Merck Sharp & Dohme Corp.
Medi1191	Solid Tumors	Modified mRNA-LPNs	Phase I	NCT03946800	MedImmuneLC
mRNA-2416	Solid Tumors and Lymphoma	Modified mRNA-LNPs	Phase I/II	NCT03323398	Moderna
mRNA-2752	Solid Tumors and Lymphoma	Modified mRNA-LNPs	Phase I	NCT03739931	Moderna
mRNA-2752	Carcinoma	Modified mRNA-LNPs	Phase I	NCT02872025	Moderna
BNT122	Colorectal Cancer Stage II/III	Modified mRNA-LPX	Phase II	NCT04486378	BioNTech
MVT-5873	Pancreatic Cancer	Not disclosed	Phase I	NCT02672917	BioNTech
BNT131	Metastatic Neoplasm	Not disclosed	Phase I	NCT03871348	BioNTech
**The Liver as a Bioreactor**					
**Encoding Cytokines**					
BNT152, BNT153	Solid Tumor	Not disclosed	Phase I	NCT04710043	BioNTech
BNT151	Solid Tumor	Not disclosed	Phase I/II	NCT04455620	BioNTech
mRNA-6231	Autoimmune Disorders	Modified mRNA-LNPs	Phase I	NCT04916431	Moderna
**Encoding Antibodies**					
BNT142	Solid Tumor	Encoding Ab	Phase I/II	NCT04503278	BioNTech
mRNA-1944	Chikungunya	Modified mRNA-LNPs	Phase I	NCT03829384	Moderna

Abbreviations: Ab—antibodies; NSCLC—non-small-cell lung cancer.

**Table 3:** mRNA Nanomedicines for Protein Replacement Therapies in Clinical Trials. A list of clinical trials for protein replacement applications is provided in this table.

mRNA Delivery for Protein Replacement Therapies					
mRNA-3927	Propionic Acidemia	Modified mRNA-LNPs	Phase 1/2	NCT04159103	Moderna
MRT5005	Cystic Fibrosis	Modified mRNA-LNPs	Phase 1/2	NCT03375047	Translate Bio

**Table 4:** mRNA Nanomedicines for Gene editing in Clinical Trials. A list of clinical trials for gene editing applications is provided in this table. Abbreviations: sg, single-guide.

Gene Editing					
NTLA-2001	Hereditary Transthyretin Amyloidosis	Cas9 mRNA and sg mRNA-LNPs	Phase 1	NCT04601051	Intellia Therapeutics
NTLA-2002	Hereditary Angioedema	Cas9 mRNA and sg mRNA LNPs	Phase 1/2	Not registered yet	Intellia Therapeutics

**Table 5:** mRNA Nanomedicines for cardiovascular diseases in Clinical Trials. A list of clinical trials for cardiovascular diseases is provided in this table.

Cardiovascular Diseases					
AZD8601	Heart Failure	Modified mRNA-LNPs	Phase II	NCT03370887	AstraZeneca
